# Laparoscopy-assisted trans-hiatal endoscopic removal of an intragastric balloon after placement-related esophageal perforation

**DOI:** 10.1055/a-2183-5963

**Published:** 2023-11-07

**Authors:** Pablo Cortegoso Valdivia, Giorgio Dalmonte, Marina Valente, Lucia Ballabeni, Federica Gaiani, Gian Luigi de' Angelis, Federico Marchesi

**Affiliations:** 1 18630 Gastroenterology and Endoscopy Unit, University Hospital of Parma, Parma, Italy; 2 18630 Unit of General Surgery, University Hospital of Parma, Parma, Italy; 3 9370 Department of Medicine and Surgery, University of Parma, Parma, Italy

Intragastric balloon placement is a minimally invasive endoscopic procedure for the treatment of obesity 1 2 . Severe adverse events such as gastric perforation, migration, and intestinal obstruction, albeit rare, may occur 3 4 ; esophageal perforation due to balloon insertion has been reported in only a handful of cases 5 .

A 29-year-old man (body mass index [BMI] 44 kg/m ^2^ ) presented with acute chest pain and abrupt onset respiratory failure during the endoscopic placement of an intragastric balloon (BioEnterics intragastric balloon [BIB]) in another hospital. He was initially treated with pleural drainage before emergent referral to our center. Computed tomography revealed the presence of the 12-cm intragastric balloon in the apex of the left pleural cavity ( Fig. 1 ), with evidence of pneumothorax and pneumomediastinum next to the lower third of the esophagus.

**Fig. 1:**
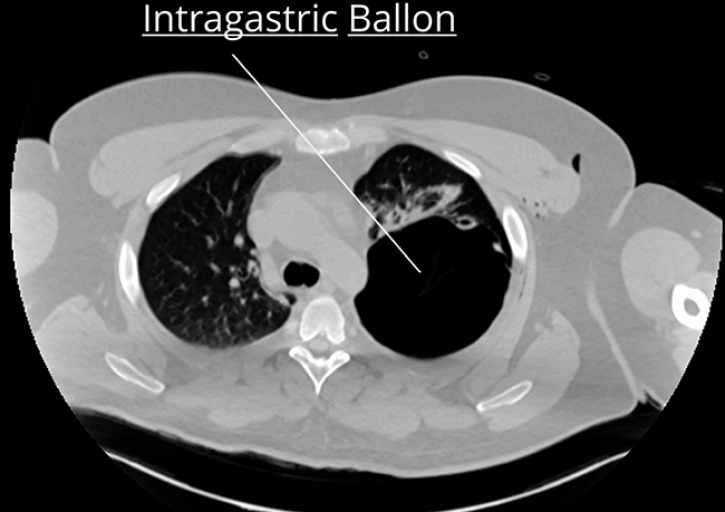
Preoperative computed tomography scan showing the intragastric balloon in the left pleural cavity.

Because of his life-threatening condition, a damage-control two-stage surgery was planned. During the first stage, a laparoscopy-assisted trans-hiatal endoscopic removal of the balloon was performed. After the abdominal cavity had been accessed, a standard gastroscope (Olympus GIF-1100) was guided through the esophageal hiatus into the mediastinum ( Fig. 2 ), where a wide esophageal laceration was observed. After the balloon had been located at the apex of the left pleural cavity ( Fig. 3 **a** ), balloon deflation was performed by needle puncture ( Fig. 3 **b** ). The deflated balloon was then grasped with a rat-toothed alligator forceps ( Fig. 3 **c** ) and dragged through the hiatus; the definitive trans-hiatal removal being performed with the help of surgical forceps ( Video 1 ).

**Fig. 2:**
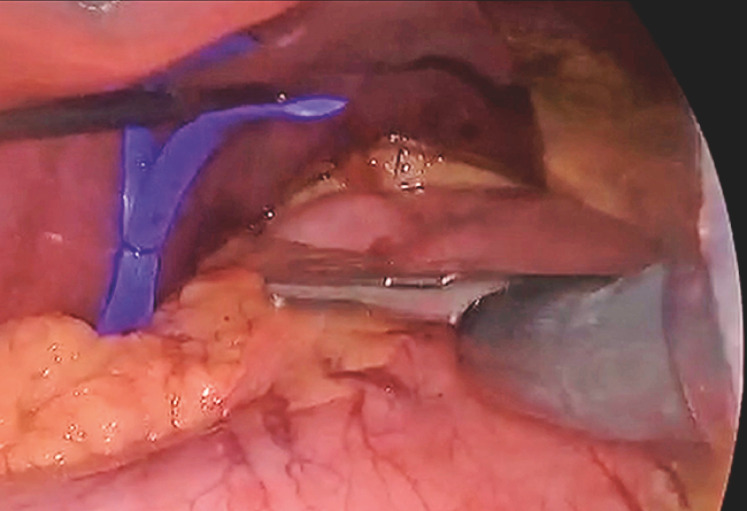
Laparoscopic view of the gastroscope entering the abdominal cavity through a 12-mm surgical port.

**Fig. 3:**
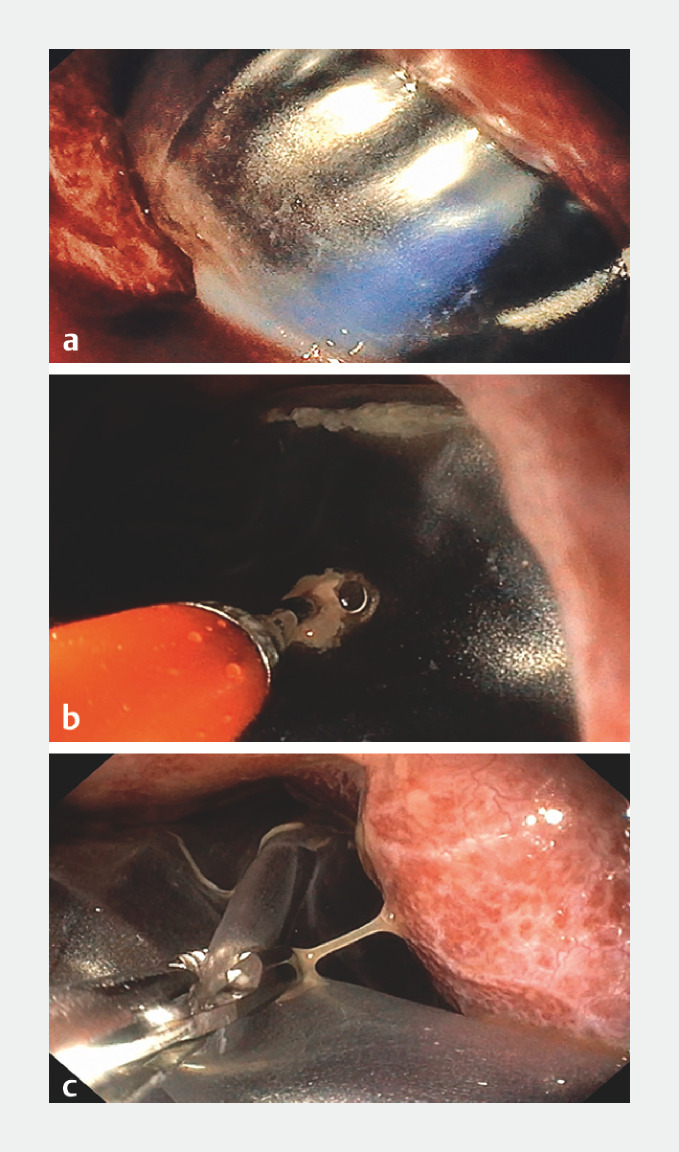
Endoscopic views showing: **a** the intragastric balloon located at the apex of the left pleural cavity; **b** the balloon being deflated by needle puncture; **c** the deflated balloon being grasped by foreign-body forceps.

Laparoscopy-assisted endoscopic removal of an intragastric balloon from the pleural cavity, after esophageal rupture during its placement.Video 1

After this, esophageal transection was performed under endoscopic control and a gastrostomy tube was placed. Once the patient had been discharged from the intensive care unit and was receiving total enteral nutrition, second-stage surgery was scheduled after a 3-month interval and a totally mini-invasive laparoscopic/thoracoscopic esophagogastric anastomosis was subsequently performed (BMI 31 kg/m ^2^ at the time of surgery).

At 6-month follow-up, the patient was in good condition and asymptomatic.

Endoscopy_UCTN_Code_CPL_1AH_2AG

## Notes

Endoscopy E-Videos https://eref.thieme.de/e-videos
*E-Videos* is an open access online section of the journal *Endoscopy* , reporting on interesting cases and new techniques in gastroenterological endoscopy. All papers include a high-quality video and are published with a Creative Commons CC-BY license. Endoscopy E-Videos qualify for HINARI discounts and waivers and eligibility is automatically checked during the submission process. We grant 100% waivers to articles whose corresponding authors are based in Group A countries and 50% waivers to those who are based in Group B countries as classified by Research4Life (see: https://www.research4life.org/access/eligibility/ ). This section has its own submission website at https://mc.manuscriptcentral.com/e-videos .
